# Site-Specific Climatic Responses of Radial Growth in *Pinus densata* in Northwestern Yunnan, Southwestern China

**DOI:** 10.3390/plants15152250

**Published:** 2026-07-23

**Authors:** Chun Tao, Tao Yan, Defen Wang, Mei Sun, Yong Zhang, Yun Zhang

**Affiliations:** 1 College of Ecology and Environment (College of Wetlands), Southwest Forestry University, Kunming 650224, China; 15154840844@163.com (C.T.); y_taoisme@163.com (T.Y.); sm0510215@163.com (M.S.); zhy930@swfu.edu.cn (Y.Z.); 2 Fuxian Lake National Wetland Park Protection and Management Center, Chengjiang 652500, China; 18087162307@163.com

**Keywords:** *Pinus densata*, tree-ring width, dendroclimatology, growth–climate relationships, southeastern Tibetan Plateau

## Abstract

*Pinus densata* is an endemic and dominant conifer in subalpine forests along the southeastern margin of the Tibetan Plateau, where it plays important ecological roles and provides high-resolution tree-ring records for assessing climate–growth relationships. However, the extent to which climatic controls on growth vary among sites with contrasting habitat conditions within the same region remains insufficiently resolved. In this study, we investigated *P. densata* at three sites in northwestern Yunnan, southwestern China: Yulong Snow Mountain (YL), Haba Snow Mountain (HB), and Shika Snow Mountain (SK). Residual tree-ring width chronologies were developed and analyzed by using response function analysis, redundancy analysis (RDA), and moving-window correlation analysis to identify key climatic factors and evaluate site-specific and temporal variation in growth–climate relationships. The results showed that radial growth responses differed markedly among sites. At YL and HB, ring width was negatively associated with mean temperature in May and positively associated with May precipitation, suggesting that early-growing-season moisture availability may play an important role under warm conditions before full monsoon onset. Warmer conditions in August generally favored growth at both sites. In contrast, radial growth at SK was positively associated with September mean temperature, suggesting a stronger association with late-growing-season thermal conditions at the higher-elevation site. RDA further retained current May mean temperature, current August mean temperature, and previous December precipitation as significant explanatory variables, indicating that radial-growth variation was linked to both current-year thermal conditions and antecedent winter moisture. Moving-window analysis showed that growth–climate relationships varied in strength and direction over time, with distinct site-specific patterns. Overall, these findings indicate that climatic responses of *P. densata* radial growth in northwestern Yunnan showed spatial heterogeneity and temporal non-stability. This study provides dendroecological evidence that site-level differences and temporal non-stability should be considered when assessing the potential responses of monsoon-influenced subalpine forests to ongoing regional warming.

## 1. Introduction

At present, global climate change is characterized by a pronounced warming trend, which is expected to profoundly and inevitably alter forest productivity, species distributional ranges, and ecosystem structure and functioning. Moreover, ongoing climate change may shift forests from net carbon sinks to net carbon sources, which would in turn reinforce the pace of global warming [1]. Under intermediate emission scenarios, global mean surface temperature is projected to increase by at least 2.1 °C by the end of the 21st century [2], and a range of climate models consistently predicts persistent warming across China [3]. With accelerating climate change, some regions may experience pronounced decreases in precipitation and increases in the frequency and severity of extreme drought [2], which can subsequently alter the spatial distribution of tree populations. Therefore, disentangling forest ecosystem responses to climate change and their driving mechanisms has emerged as a core frontier in contemporary global-change ecology.

Through physiological regulation, trees integrate specific climatic signals into variations in ring width, effectively converting climate forcing into a measurable growth record. Owing to their annual resolution, continuity, and sensitivity to climatic variability [4,5], tree rings constitute a key proxy archive for forest ecological and paleoclimatic studies. In recent decades, tree-ring records have been widely leveraged to characterize climate variability and to probe ecosystem response mechanisms from regional to global scales. For instance, a global synthesis spanning more than 2700 sites showed that tree rings could support both “retrospective” climate reconstructions and “prospective” constraints on how ecosystems may respond to future warming [6]. Using global tree-ring datasets, Wilmking et al. [7] systematically quantified growth–climate linkages and showed that these relationships varied strongly across bioclimatic zones and taxa, and could be non-stable over time. At the regional scale, Wei et al. [8] used *Pinus tabuliformis* tree-ring series from Jigong Mountain in China’s north–south transition zone, and found a strong sensitivity of radial growth to growing-season temperatures and to moisture conditions in preceding periods. In addition, bibliometric evidence indicated that dendrochronology was shifting from predominantly correlation-based approaches to mechanistic inference and multi-source data synthesis [9].

Radial growth reflects the cyclical activity of vascular cambium cells and is jointly regulated by environmental forcing (e.g., climate variability) and intrinsic physiological attributes (e.g., tree size) [10]. Under climate change, temperature and precipitation are widely recognized as the primary climatic controls on vegetation growth. At high elevations, cold conditions restrict cambial cell division and xylem development, thereby making temperature a principal control on tree growth. In contrast, at lower elevations, stronger evaporative demand combined with limited precipitation often renders water stress the primary constraint on tree growth [11]. When climatic stress surpasses a tree’s ecological tolerance, persistent constraints (e.g., water deficit or low temperature) can prematurely shut down cambial activity, shortening the growing season and reducing carbon allocation to radial growth, which may produce narrow rings or even missing rings [10]. Conversely, when conditions become more favorable, cambial division and differentiation can resume, and xylem development may exhibit adaptive anatomical adjustments, including distinctive ring features. These anatomical variations encode climatic variability while also revealing tree survival strategies expressed through coordinated physiological regulation and structural plasticity.

The Tibetan Plateau is often regarded as both a climate change “hotspot” and a global warming “amplifier” [12,13], and its ecosystems respond strongly to climatic variability, with even modest shifts capable of producing substantial ecological effects. Located in the core of the Hengduan Mountains at the southeastern margin of the Tibetan Plateau, the northwestern Yunnan Plateau serves as a key pathway for Indian monsoon moisture to penetrate the Plateau. This region retains relatively intact ecosystems and exceptionally rich vegetation types and biodiversity, is highly sensitive to global climate change, and thus provides an ideal natural laboratory for tree-ring–climate studies. Under such regional warming, trees in high-elevation monsoon regions may experience multiple climatic stresses, including increased evaporative demand before the full onset of summer rainfall, intensified early-growing-season moisture stress, and altered cold-season moisture storage or snow-related legacy effects [2,13,14]. In addition, shifts in seasonal thermal conditions may affect cambial activity, xylem formation, and the timing or length of the effective growing period [10]. Over the past decades, extensive dendroclimatological work across the northwestern Yunnan Plateau and the central Hengduan Mountains has documented pronounced post–Little Ice Age warming together with shifts in regional moisture regimes [15,16]. Research on dominant high-elevation conifers, including *Abies georgei* [17], *Picea likiangensis* [18], and *Larix potaninii* [19], has demonstrated striking interspecific contrasts in climate sensitivity. However, even within a single region, the dominant controls on growth may vary widely across species because of niche differentiation and physiological traits, reflecting species-specific pathways of ecological adaptation. For instance, within the same mountain system, *A. georgei* growth was primarily constrained by summer temperatures, whereas *P. likiangensis* and *Pinus densata* were more responsive to growing-season water availability [20,21,22]. Therefore, broad-scale comparative analyses of intraspecific responses across regions are essential for robust assessments of forest vulnerability and stability under projected future climates.

*P. densata* is an endemic conifer of the alpine–subalpine belt in southwestern China, primarily distributed in western Sichuan, northwestern Yunnan, and southeastern Tibet [23]. The species typically occurs at 2600–3500 m a.s.l. [24], exhibits strong ecological adaptability, including tolerance to cold and drought, and often forms pure or mixed forests on slopes and in shallow, stony soils [25,26]. As a dominant species in the subalpine conifer belt of northwestern Yunnan, *P. densata* contributes substantially to regional carbon storage, soil retention, and watershed regulation, while its annual rings provide high-resolution growth–environment proxies for assessing climatic controls on tree growth over recent decades [27]. Previous dendrochronological studies have shown that radial growth of *P. densata* in northwestern Yunnan is sensitive to seasonal hydrothermal conditions, especially early-growing-season moisture deficits, and may also be influenced by climatic legacies from the preceding winter [21,28]. Although these studies have provided important evidence for the climatic responses of *P. densata*, comparisons of site-specific climate sensitivity within the same region remain limited. In particular, whether the relative importance of these climatic controls changes systematically among sites with contrasting elevation and local habitat conditions remains unclear. In addition, it is still unknown whether these growth–climate relationships have remained stable under recent regional warming.

In this study, we developed site-level tree-ring width chronologies from Yulong Snow Mountain (YL), Haba Snow Mountain (HB), and Shika Snow Mountain (SK) to examine the site-specific climate–growth relationships of *P. densata* in northwestern Yunnan. Specifically, we aimed to identify key climatic factors associated with radial growth, compare site-specific growth–climate relationships among the three sites, and evaluate the temporal stability of these relationships. We hypothesized that (1) radial growth at the relatively lower-elevation YL and HB sites would be more sensitive to hydrothermal conditions, particularly moisture limitation intensified by warm conditions before full monsoon onset, whereas growth at the higher-elevation SK site would show a stronger temperature-related response because of greater thermal limitation near the upper distribution range; and (2) these growth–climate relationships would show temporal non-stability under ongoing regional warming.

## 2. Results

### 2.1. Statistical Characteristics of the Chronologies

As summarized in Table 1, the residual ring-width (RES) chronologies spanned 87–197 years, with the earliest record dating back to 1819. For the SK chronology, sample replication was relatively low in the early portion of the chronology. According to the ARSTAN output and sample-code records, before 1900, the SK RES chronology was represented by up to 14 trees and 22 cores. Because sample replication was relatively low in the early portion of the SK chronology, all climate–growth analyses were conducted over the common and better-replicated period of 1953–2015. The ring-width index (RWI) ranged from 0.64 to 1.36, reaching a maximum in 1884 (1.36) and a minimum in 1915 (0.64). The expressed population signal (EPS) at all sites exceeded the conventional threshold of 0.85, indicating that the core samples captured a coherent common growth signal of *P. densata* across the study area. In addition, the mean sensitivity (MS = 0.08–0.14) and standard deviation (SD = 0.07–0.12) indicated detectable interannual variability in radial growth. Notably, signal-to-noise ratios (SNRs) were consistently high, with the SK chronology reaching 30.04, which reflected comparatively low non-climatic noise and a clear expression of the common signal. Moreover, the first principal component (PC1) explained 44.62% of the variance in the HB chronology, which was higher than that in YL and SK. Overall, these statistics indicated that all three chronologies were of high quality and suitable for subsequent dendroclimatological analyses.

### 2.2. Radial Growth Responses to Climatic Factors

**Figure 1:**
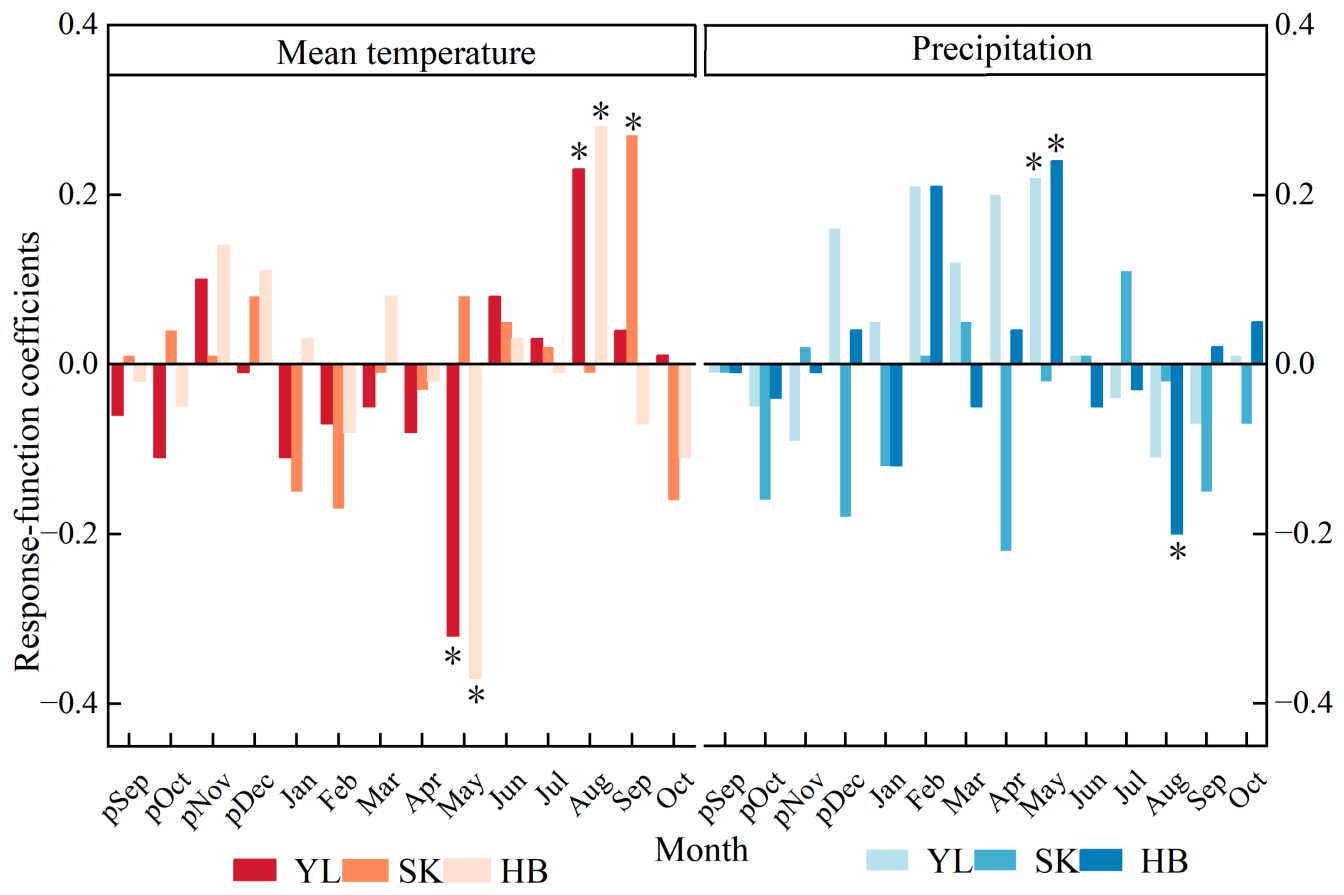
Monthly response-function coefficients between residual chronologies and climatic variables from previous September to current October during 1953–2015. Months prefixed with “p” indicate months of the previous year, for example, pDec represents December of the previous year, whereas months without this prefix indicate months of the current year. Asterisks indicate significant coefficients at *p* < 0.05 based on bootstrap resampling in DendroClim2002.

Response function analysis (Figure 1) showed that the radial growth of *P. densata* differed markedly among the three sites in its responses to climatic factors. At YL, climate sensitivity was concentrated in the early and main growing seasons: ring width was significantly negatively correlated with the mean temperature in May of the current year (*p* < 0.05), significantly positively correlated with precipitation in May (*p* < 0.05), and significantly positively correlated with the mean temperature in August of the current year (*p* < 0.05). In contrast, the climate response at SK was relatively concentrated, with growth mainly influenced by temperature variability in the late growing season and showing a significant positive correlation only with the mean temperature in September of the current year (*p* < 0.05). The response pattern at HB was more complex, showing clear seasonal differentiation and coupled hydrothermal controls: ring width was significantly negatively correlated with temperature in May and positively correlated with precipitation in May, whereas it was significantly positively correlated with temperature in August and negatively correlated with precipitation in August (all *p* < 0.05).

Considering the seasonality of tree growth, we conducted multi-month composite response-function analyses for the growing-season months (Table 2). The results showed that radial growth responded significantly to climatic conditions mainly in spring and summer. At YL and HB, growth was significantly negatively correlated with mean spring temperature and significantly positively associated with spring precipitation, whereas no significant relationships with spring climate were detected at SK. In summer, the spatial heterogeneity in climate sensitivity became more pronounced: only HB showed significant relationships with seasonal climate, showing a positive correlation with mean summer temperature and a negative correlation with summer precipitation, whereas no significant summer relationships were observed at YL or SK.

### 2.3. Redundancy Analysis Between Residual Chronologies and Climatic Variables

**Figure 2:**
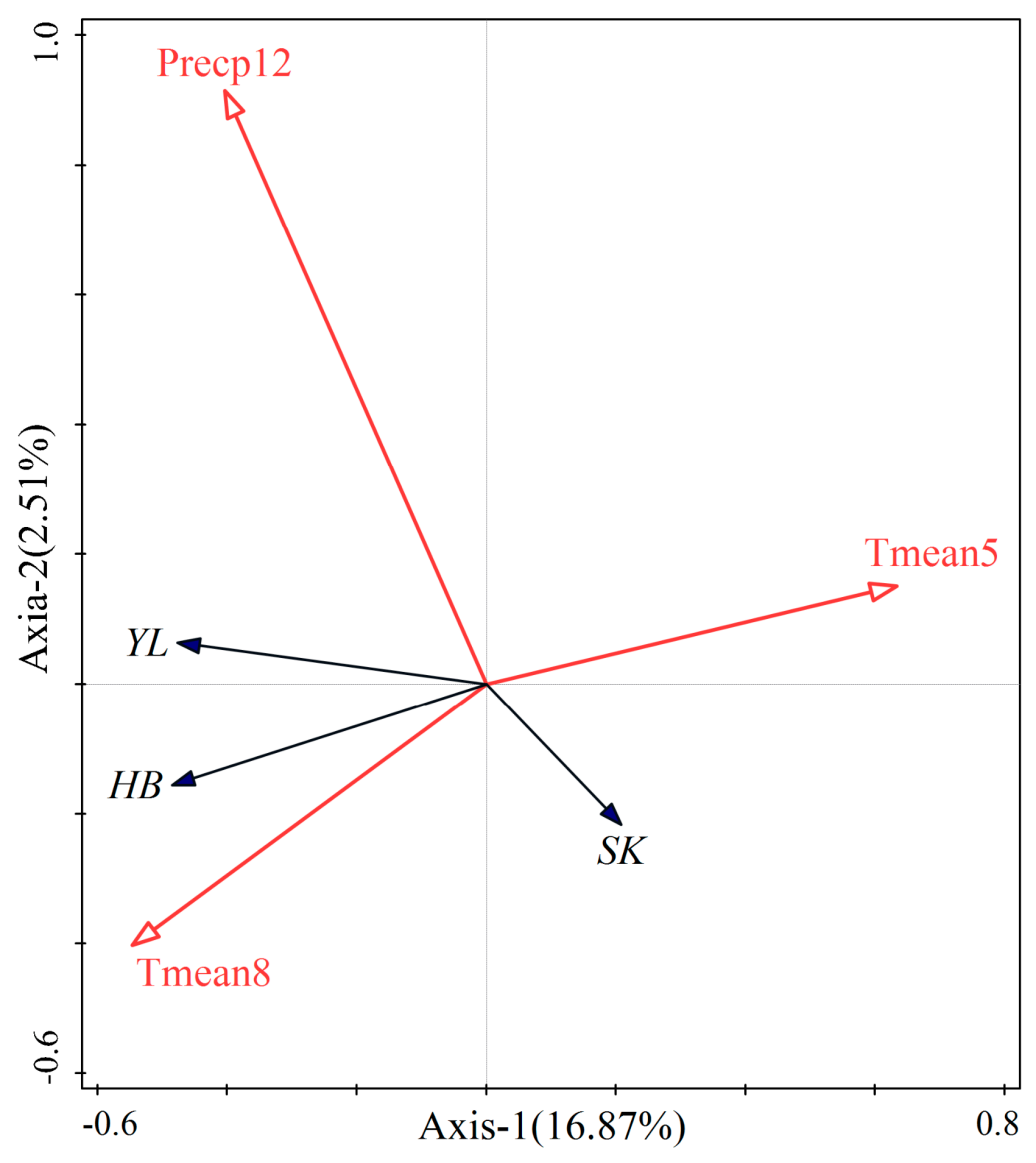
Redundancy analysis (RDA) between the residual chronologies and climatic variables (1953–2015). Only significant (*p* < 0.05) climatic variables were presented. The longer the vector of a climate variable (red line), the greater its contribution. Black lines represent the residual chronologies of the three sampling sites. The cosine of the angle between the climate vector and the chronology vector represents the correlation coefficient between the chronology and the climate factor. Vectors pointing in the same direction indicate a positive correlation, whereas vectors pointing in opposite directions indicate a negative correlation. Tmean5, mean temperature in current May; Tmean8, mean temperature in current August; Precp12, precipitation in previous December.

As shown in Figure 2, among the 28 monthly climatic variables considered from the previous September to the current October, RDA with forward selection retained current May mean temperature, current August mean temperature, and previous December precipitation as significant explanatory variables. Together, these variables accounted for 19.4% of the total variation in the three residual chronologies, with an adjusted explained variation of 15.3% (Table S1). Among the selected variables, previous December precipitation was positively associated with radial growth at YL and HB, but negatively associated at SK. Current May mean temperature showed contrasting relationships among sites, with negative associations with ring width at YL and HB, but a positive association at SK. Current August mean temperature also showed contrasting relationships, being positively associated with ring width at YL and HB, but negatively associated at SK. Overall, the RDA results were broadly consistent with the response function analysis and further indicated that radial growth of *P. densata* was associated with current-year thermal conditions and antecedent winter precipitation.

### 2.4. Temporal Variation in Growth–Climate Relationships

**Figure 3:**
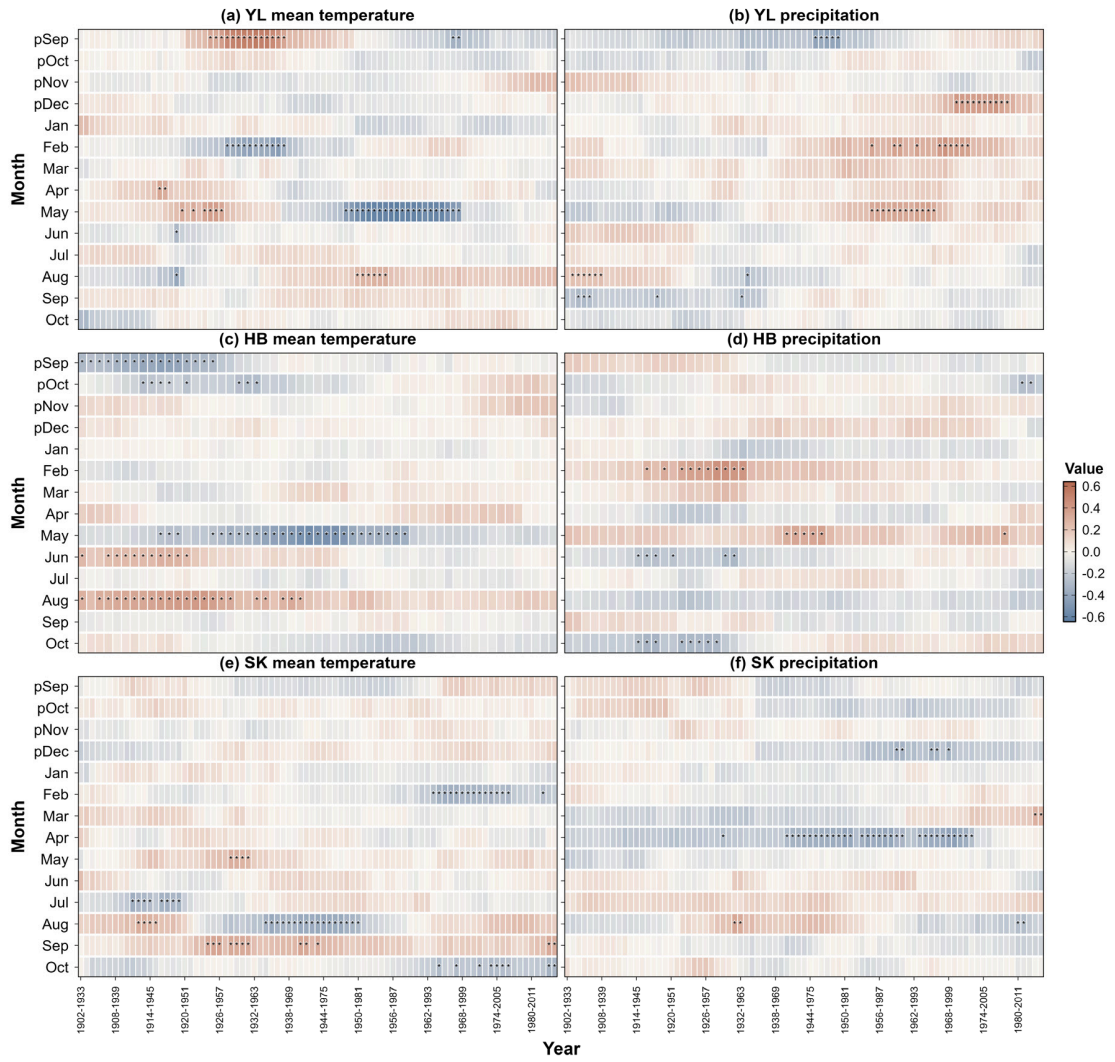
Moving-window response analysis between residual chronologies and climate factors. The year on the x-axis represents the ending year of each 32-year moving window. Months prefixed with “p” indicate months of the previous year; for example, pDec represents December of the previous year, whereas months without this prefix indicate months of the current year. Asterisks indicate significant response coefficients at *p* < 0.05.

The results of moving-window analysis were generally consistent with those of response function analysis, but the temporal stability of the growth–climate relationships differed among sampling sites (Figure 3). At YL, the effect of May temperature on *P. densata* growth showed a phased change. May temperature was positively correlated with radial growth in the early moving windows, with significant positive correlations appearing in several windows ending in the 1950s. In later windows, this relationship gradually shifted to a negative one, and significant negative correlations were mainly observed in windows ending during 1980–2000. Meanwhile, the relationship between May precipitation and radial growth changed from negative to positive over time, with significant positive correlations mainly occurring in windows ending during 1987–1998.

At HB, radial growth showed relatively stable responses to climatic factors. May temperature was generally negatively correlated with radial growth, whereas August temperature was generally positively correlated with radial growth. Significant negative correlations with May temperature were mainly observed in windows ending during 1978–2000, while significant positive correlations with August temperature were concentrated in windows ending during 1963–1988.

At SK, September temperature was positively correlated with radial growth in most moving windows, with significant positive correlations occurring intermittently, mainly in windows ending from the mid-1950s to the early 1970s. Significant positive correlations also appeared in the final two windows ending in 2014 and 2015. In addition, April precipitation showed a relatively stable negative relationship with radial growth, with significant negative correlations mainly observed in windows ending during 1971–2003.

## 3. Discussion

The response function and RDA results indicated that temperature and precipitation in May and August, together with precipitation in the previous December, were key climatic variables associated with *P. densata* radial growth in this region. Growth–climate response patterns were more similar between YL and HB, possibly because the two sites were geographically closer, whereas climatic sensitivity at SK appeared more concentrated in late-season temperature. These findings were broadly consistent with our expectation that radial growth of *P. densata* exhibited site-specific climatic sensitivity, while further indicating that growth was jointly regulated by both temperature and precipitation under regional warming and large interannual precipitation variability. Moreover, moving-window analysis indicated that the temporal stability of these growth–climate relationships varied among sites.

### 3.1. Response Characteristics of P. densata Radial Growth to Climatic Factors

Both the response function and RDA results indicated that high May temperatures were associated with reduced radial growth at YL and HB, whereas increased precipitation during the same period was associated with enhanced growth. These patterns suggest that radial growth was closely associated with early-growing-season hydrothermal conditions at these two sites. From early spring to early summer, warming without a corresponding increase in rainfall may increase evapotranspiration and accelerate soil moisture depletion, thereby lowering plant water status, reducing stomatal conductance, and constraining photosynthetic carbon uptake. Such conditions may delay cambial reactivation and restrict earlywood cell division, ultimately limiting radial growth [29,30]. By contrast, greater May precipitation may alleviate early-season moisture deficits, maintain more favorable plant water status, and promote cell enlargement. Spring moisture availability has been widely regarded as an important control on xylem formation [31], and similar results have been reported for *P. yunnanensis* on the northwestern Yunnan Plateau [32]. At SK, near the upper elevational limit, growth showed comparatively weak sensitivity to May hydrothermal conditions, which may indicate that cambial onset at this site was more strongly constrained by low-temperature thresholds than by early-season moisture availability [10,33].

During the growing season, warmer August conditions were positively associated with radial growth at YL and HB. Moderate warmth during this period may enhance enzymatic activity, photosynthetic rates, and cell development, thereby supporting carbohydrate accumulation and wider xylem increments, consistent with findings for other high-elevation conifers [22,34]. At HB, however, August precipitation was negatively associated with growth, suggesting that excessive rainfall during this period may not favor radial growth. One possible explanation is that persistent rainfall increases cloudiness and reduces incoming solar radiation, thereby limiting heat supply and carbon assimilation during active xylem development. It is also possible that sustained wet conditions temporarily increase soil moisture excess in some microsites and constrain root activity [35]. By contrast, the August hydrothermal signal was weaker at SK. Comparable site-to-site divergences in growth sensitivity have also been reported for subalpine *P. yunnanensis* [36] and other conifers [37], suggesting that differences in climatic sensitivity among sites may reflect the combined effects of elevation, topography, and local habitat conditions.

At SK, the climatic signal was more concentrated, with ring width responding primarily to mean temperature in September and only weakly to spring–summer climate. This pattern suggests that radial growth at the higher-elevation site was more responsive to late-growing-season thermal conditions. Warmer September conditions may prolong the period of carbon assimilation and support the completion of secondary xylem development [38,39]. In addition, because SK is located at a higher elevation, thermal limitation may remain more important than moisture limitation during the late growing season, and autumn warming may help relieve late-season cold stress [33,40]. In contrast, at the relatively lower-elevation YL and HB sites, summer thermal conditions may already be sufficient and much of radial growth may be largely completed by August, reducing sensitivity to September temperature. Similar positive effects of late-growing-season temperature on radial growth have been reported for *Pinus wallichiana* in the Everest region of the Tibetan Plateau [41] and *Abies fargesii* on the southern slopes of the Qinling Mountains [42].

### 3.2. Key Hydrothermal Drivers Revealed by RDA and Their Ecological Implications

In the RDA, current-year May and August mean temperatures explained a significant portion of radial-growth variability across the three sites and were broadly consistent with the response function results, suggesting that RDA effectively captured the major hydrothermal signals associated with tree growth. However, the two approaches did not identify exactly the same climatic variables. In particular, RDA additionally highlighted precipitation in the previous December, indicating that antecedent winter conditions may also influence radial growth. This difference likely reflects the distinct statistical properties of the two methods rather than contradictory ecological interpretations. Similar method-dependent differences have also been reported in tree-ring studies from the central Hengduan Mountains and around Lugu Lake [28,43]. Taken together, the response function analysis and RDA provide complementary evidence that radial growth of *P. densata* in northwestern Yunnan was jointly influenced by early-growing-season hydrothermal conditions, late-growing-season temperature, and antecedent winter precipitation.

The positive association between previous December precipitation and growth at YL and HB may reflect beneficial effects of winter moisture on subsequent growing-season water availability. In high mountains influenced by the monsoon, greater winter precipitation, often in the form of snowfall, may increase snow cover or cold-season soil moisture and contribute to spring moisture recharge, thereby favoring radial growth in the following year [18,44,45,46]. By contrast, the contrasting response at SK suggested that antecedent winter precipitation did not operate uniformly across sites. Because this study did not directly quantify snowpack dynamics, soil moisture storage, or soil depth, the mechanisms underlying this site difference should be interpreted cautiously. It is possible that elevation, local substrate conditions, and the balance between moisture supply and thermal limitation jointly mediated the effect of winter precipitation on growth. Overall, the contrasting site responses further indicated that antecedent climatic effects on *P. densata* growth were context-dependent within northwestern Yunnan.

### 3.3. Temporal Stability of Growth–Climate Relationships

The moving-window analysis indicated temporal non-stability in the relationships between radial growth of *P. densata* and climatic factors at YL, suggesting that climatic controls on growth changed through time rather than remaining constant. This result was consistent with the growing recognition in dendroclimatology that climate–growth relationships can vary across time, species, regions, and even sites within the same region [6,7,47]. Similar temporal variation has also been reported for major conifer species on Shika Snow Mountain, for *P. densata* on Baima Snow Mountain, and for *P. densata* in Milin [20,21,48]. Together, these comparisons suggest that temporal changes in growth–climate relationships may be a common feature of high-elevation conifers in this broader mountain region.

**Figure 4:**
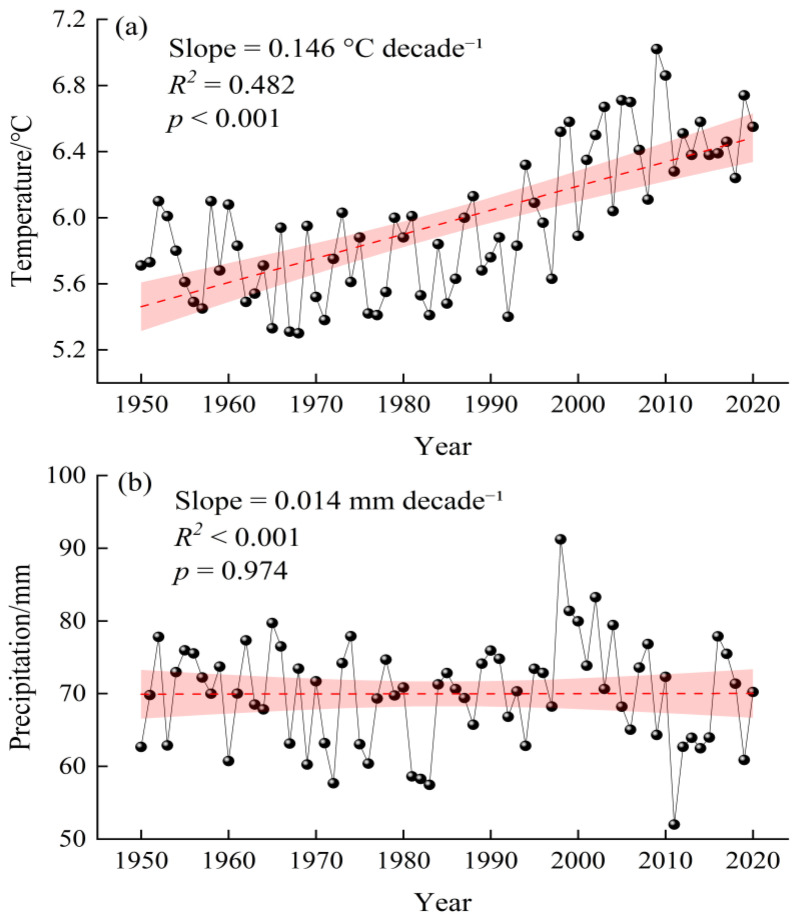
Interannual trends in mean annual temperature (**a**) and annual precipitation (**b**) in the study area from 1950 to 2020. Red dashed line indicates the linear trend; shaded band indicates the 95% CI.

The temporal instability of climate–growth relationships may partly reflect changes in regional hydrothermal conditions during recent decades. In the study area, mean annual temperature increased significantly during 1950–2020, with accelerated warming after the mid-to-late 1980s, whereas annual precipitation showed strong interannual variability without a significant long-term linear trend (Figure 4). Such warming may have increased evaporative demand before the full onset of the summer monsoon, thereby strengthening early-growing-season moisture limitation at the relatively lower-elevation YL and HB sites [29,30]. The result was consistent with the shift in the May temperature response at YL from positive in earlier moving windows to negative in later windows, together with the increasingly positive response to May precipitation. In addition, interannual variability in precipitation under the monsoon-influenced climate may have altered soil moisture availability and further contributed to temporal changes in growth responses [49]. However, because stand-level soil moisture, cambial phenology, and physiological measurements were not available, these explanations should be regarded as plausible mechanisms rather than direct evidence.

### 3.4. Ecological Implications and Management Recommendations Under Regional Warming

Under continued regional warming, *P. densata* in northwestern Yunnan may be increasingly exposed to warm–dry conditions during the early growing season, which could impose stronger constraints on radial growth [14]. At the relatively lower-elevation YL and HB sites, the negative effect of May temperature together with the positive effect of May precipitation suggests that growth was particularly sensitive to the balance between warming and moisture supply before full monsoon onset. If spring warming advances more rapidly than seasonal rainfall, evaporative demand may increase and early-season moisture stress may intensify, thereby constraining cambial activity and earlywood formation. At the same time, the positive association between August temperature and radial growth at YL and HB indicates that moderate summer warmth may still favor growth, although this positive effect is unlikely to be uniform under all conditions. When warmer conditions are accompanied by excessive rainfall or more frequent climatic extremes, the effects of temperature and precipitation on growth may become more complex. The contrasting responses among YL, HB, and SK further suggest that growth responses under continued regional warming may differ among sites, partly because local habitat conditions may mediate tree responses to changing hydrothermal regimes. These findings do not provide direct predictions of future growth dynamics, but they indicate that spatial divergence and temporal non-stability in growth–climate relationships should be considered when assessing the potential responses of *P. densata* forests to ongoing climate change. In particular, the period from May to August appears to be a climatically sensitive phase for radial growth, although the main climatic constraint may differ among sites. From a management perspective, this pattern suggests the value of site-specific monitoring of spring moisture conditions, summer thermal variability, and antecedent winter precipitation, rather than assuming a uniform climate response across the region. More broadly, our results indicate that climate warming in monsoon-influenced subalpine forests may be associated not only with changes in the strength of growth limitation, but also with shifts in the seasonal timing and site dependence of that limitation.

Future studies should further test these interpretations by integrating tree-ring records with stand-level environmental and physiological observations. In particular, long-term monitoring of local microclimate, soil moisture, snowpack conditions, cambial phenology, and plant water status would help clarify the mechanisms underlying site-specific growth responses. In addition, combining tree-ring width with complementary indicators such as wood anatomical traits, stable isotopes, basal area increment, and remote-sensing observations may provide a more comprehensive assessment of how *P. densata* forests respond to ongoing regional warming.

## 4. Materials and Methods

### 4.1. Study Area

**Figure 5:**
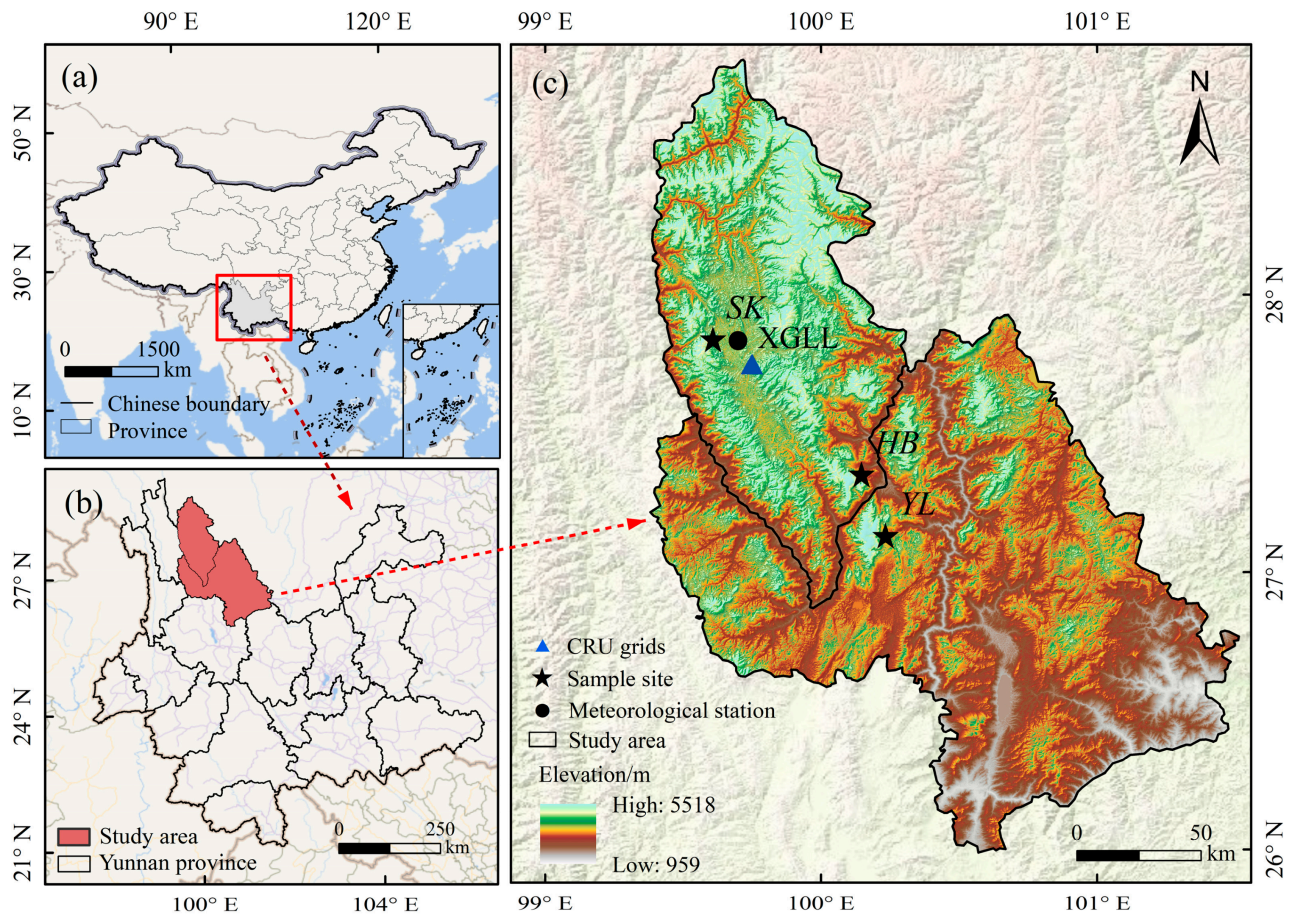
Locations of the sampling sites, CRU grid points, and nearby meteorological stations. (**a**) Location of the study area in China; (**b**) topography of Yunnan Province and the extent of the study area; (**c**) detailed distribution of the sampling sites, CRU grid points, and the Shangri-La meteorological station (XGLL).

The study area is located on the northwestern Yunnan Plateau within the core region of the Hengduan Mountains of southwestern China (26°52′–28°16′ N, 99°43′–100°12′ E) (Figure 5). The terrain is highly dissected, with nearly 6000 m of relative relief; deeply incised valleys and perennially snow-covered summits form a typical alpine–gorge landscape and create sharp vertical environmental gradients [50], providing favorable conditions for climate change research. Sampling was conducted at Yulong Snow Mountain (YL; Lijiang) and at Shika (SK) and Haba (HB) snow mountains in Shangri-La (Diqing Tibetan Autonomous Prefecture). These sites encompass well-preserved core habitats of *P. densata* and are representative of the structure and diversity of Hengduan alpine conifer forests and alpine ecosystems. The sampling plots were located in the montane conifer belt at YL, HB, and SK (3052–3623 m a.s.l.), all situated within the middle to upper elevational range of the species’ natural distribution.

**Figure 6:**
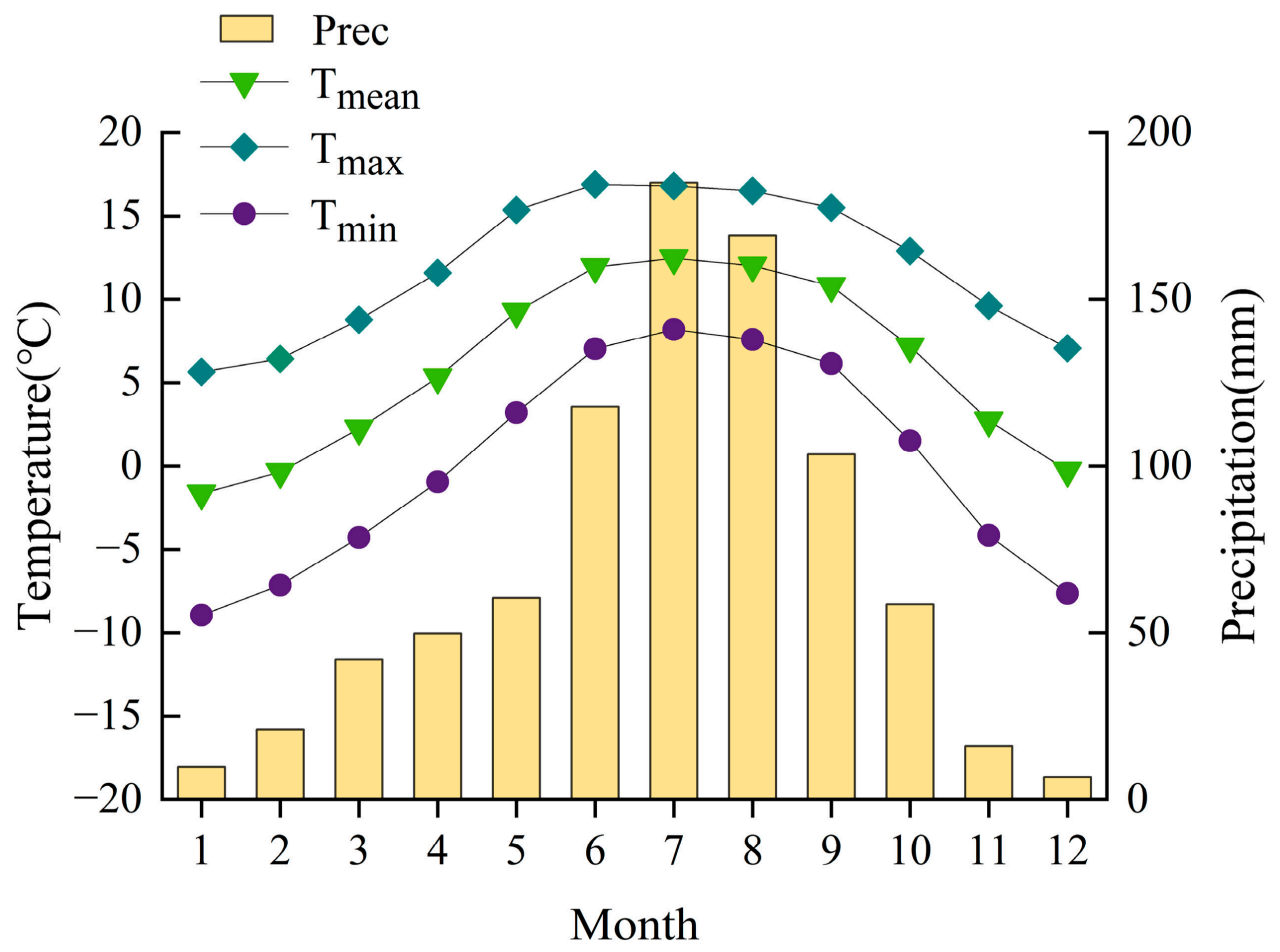
Monthly climate characteristics of the study area from 1950 to 2020. Prec: monthly mean precipitation; T_min_: monthly mean minimum temperature; T_mean_: monthly mean temperature; T_max_: monthly mean maximum temperature.

The regional climate is shaped by the South Asian summer monsoon, the East Asian monsoon, and plateau-scale thermal and dynamical forcing, resulting in a low-latitude plateau monsoon regime with warm–humid summers, cold–dry winters, and pronounced wet–dry seasonality [49]. As a windward pathway for South Asian monsoon moisture and a hotspot for plateau–monsoon interactions, the region is frequently considered a key “indicator belt” for tracking regional climate change [51]. CRU gridded climate data for 1950–2020 (Figure 6) indicate a regional mean temperature of approximately 5.97 °C, with a unimodal annual temperature cycle. January is the coldest month (−1.64 °C) and July the warmest (12.47 °C), consistent with the cold winters and cool summers typical of the plateau monsoon climate. Mean annual precipitation totals 839.65 mm, with approximately 575.38 mm occurring in June–September (68.53% of the annual total). Interannual analysis (Figure 4a) shows a significant warming trend during 1950–2020, at a rate of approximately 0.0146 °C·yr^−1^. Warming accelerated after the mid-to-late 1980s (e.g., approximately 0.0254 °C·yr^−1^ for 1986–2020), and temperatures in most years after 2000 were consistently above the mean for the 1950s–1970s. In contrast, annual precipitation showed pronounced interannual fluctuations without a significant long-term linear trend (Figure 4b), with wet and dry years alternating.

Driven by steep elevational climatic gradients and complex relief, vegetation shows pronounced vertical zonation, yielding an alpine–subalpine belt system dominated by montane conifer forests, with associated shrublands and alpine meadows. While elevational vegetation zonation is similar across YL, HB, and SK, the elevational distribution of *P. densata* varies among the sites. *P. densata* is primarily distributed at 2800–3500 m at YL, spans a broader range at HB (2000–3500 m), and reaches a higher upper limit at SK, typically occurring at 2700–3700 m. Soils at YL are mainly loam- and clay-textured and shift upslope from clay/clay loam to sandy clay, sandy loam, and loam [52]. At HB (1800–3450 m), soils are predominantly loamy, with soil types shifting from red to yellow and then to brown soils; above 3900 m, cold alpine zones are mainly characterized by gray podzolic soils and meadow soils [53]. At SK, soils in the subalpine conifer belt are primarily dark-brown soils, podzolized (bleached) soils, and alpine meadow soils. In addition to these general soil types, local edaphic conditions may differ among the sampled stands because of variation in elevation, topography, and slope position.

### 4.2. Tree-Ring Sampling and Processing

Field surveys were conducted in 2015 and 2017 at Yulong Snow Mountain, Shika Snow Mountain, and Haba Snow Mountain. Following the standards of the International Tree-Ring Data Bank (ITRDB) [54], and after considering topography, slope, and aspect, we selected one sampling site within the core distribution area of *P. densata* at each mountain. To minimize the influence of non-climatic factors on radial growth, we preferentially sampled older, vigorous dominant trees that had experienced minimal human disturbance and weak inter-tree competition. We extracted increment cores at breast height (approximately 1.3 m) using a 5.15 mm diameter increment borer, ensuring that each core included both bark and pith. At least 28 trees were sampled at each site, with two cores taken from different directions of each tree, and cores were stored in plastic tubes and labeled. In total, 186 cores from 94 trees were collected in the field, and the maximum tree age was 205 years (Table 3).

After returning to the laboratory, samples were processed following standard dendrochronological procedures. Cores were air-dried and mounted in wooden holders using white glue; after complete drying, they were progressively sanded with 120-, 400-, and 600-grit sandpaper until ring boundaries were clearly visible. Visual cross-dating was performed under a stereomicroscope, and then cores were scanned to obtain high-resolution images using an EPSON Expression 11000XL scanner (Seiko Epson Corporation, Suwa, Japan). Scanning was performed in professional mode (24-bit color) at 3200 dpi, and ring widths were measured for each core using CDendro and CooRecorder (version 9.8.4; Cybis Elektronik & Data AB, Saltsjöbaden, Sweden) with a measurement precision of 0.001 mm. We used COFECHA (version 6.06P) [55] to statistically verify cross-dating and to identify and correct dating and measurement errors, and we excluded samples that deviated strongly from the master series or contained excessive outliers to ensure accurate annual dating. Ultimately, 142 cores from 76 trees were retained for chronology development.

**Figure 7:**
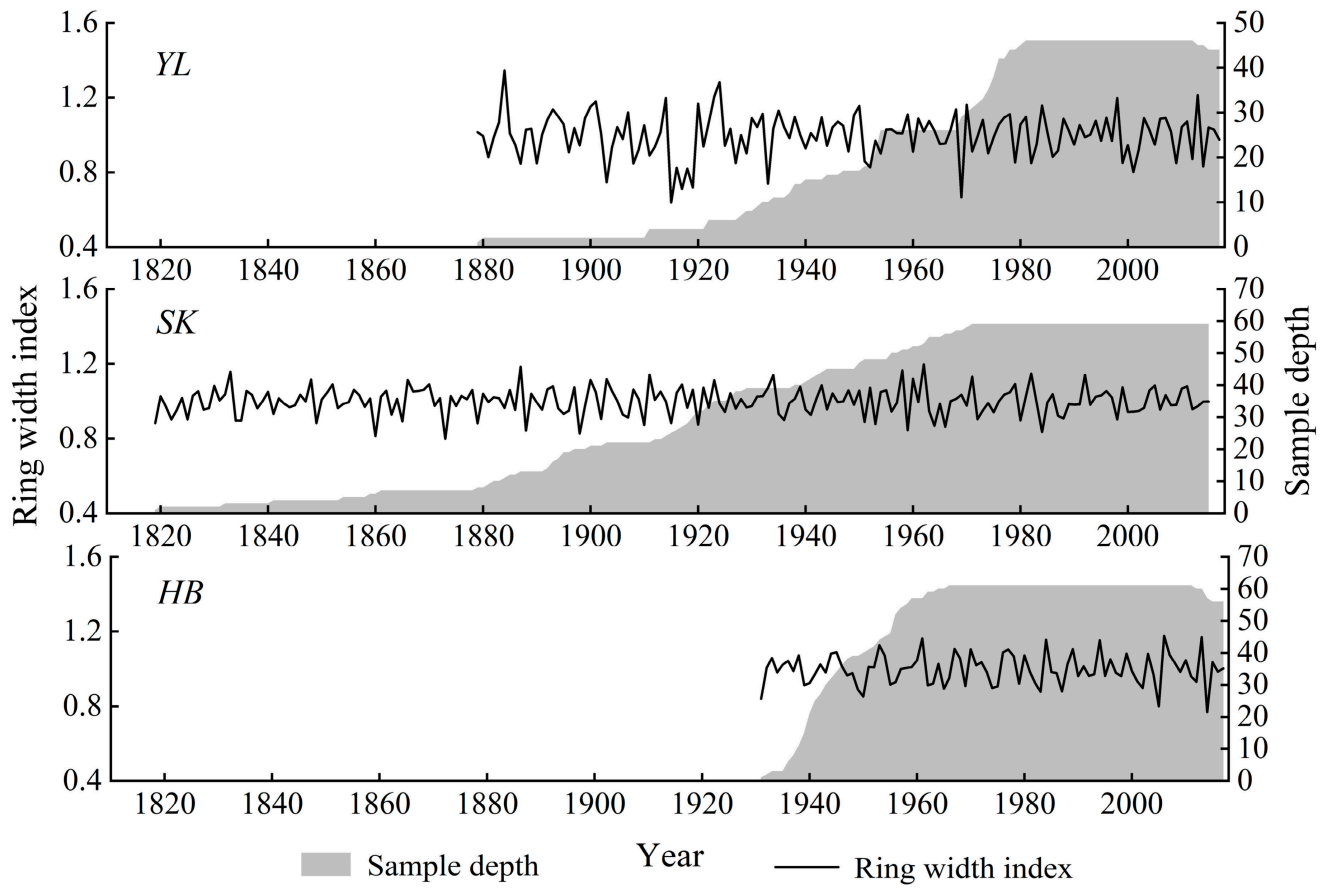
Residual tree-ring width chronology.

Ring-width series were detrended in ARSTAN (version 6.04P) [56] by using a cubic smoothing spline (67% frequency-response cutoff) to reduce non-climatic variance attributable to intrinsic growth traits and stand competition, while preserving growth signals related to climate variability. Three chronology types were produced: standard (STD), residual (RES), and autoregressive (ARS). Because the RES chronology (Figure 7) substantially reduced growth autocorrelation and retained higher-quality high-frequency climate signals, we used it for all subsequent analyses. The RES chronologies were checked for remaining autocorrelation using ARSTAN diagnostics, which showed that lag-1 autocorrelation was substantially reduced after residual chronology development.

### 4.3. Climate Data

We used monthly climate data from the CRU TS v4.06 high-resolution gridded dataset developed by the Climatic Research Unit (CRU), University of East Anglia (https://crudata.uea.ac.uk/cru/data/hrg/; accessed on 21 July 2026), at a 0.5° × 0.5° spatial resolution [57]. The CRU TS dataset spans from 1901 onward, and monthly climate data from 1901 to 2020 were extracted in this study. The variables included monthly mean temperature (T_mean_) and monthly precipitation (Pre). To describe the regional climate background and interannual climate trends, the period 1950–2020 was used. Because continuous long-term meteorological observations are limited at the high-elevation sampling sites in this mountainous region, we used the CRU-derived Shangri-La climate series to represent regional interannual climate variability. The monthly temperature and precipitation series were extracted from the same CRU grid cell centered at 27.75° N, 99.75° E, covering 27.50–28.00° N and 99.50–100.00° E, and were used for all three sampling sites. The approximate distances from the grid-cell center to YL, HB, and SK were 83.9, 59.1, and 17.0 km, respectively. Given the complex terrain, strong elevational gradients, and varying distances between the sampling sites and the CRU grid-cell center, this regional gridded climate series may not fully capture site-level microclimatic differences among the sampling sites. In addition, the CRU data were not validated against local station records in this study. Therefore, the climate–growth relationships were interpreted as responses to regional climate variability rather than direct measurements of local microclimate at each stand.

### 4.4. Statistical Analysis

Relationships between radial growth and climate were first evaluated using response function analysis implemented in DendroClim2002 [58]. The residual (RES) chronology from each site was used as the growth variable, and monthly mean temperature and precipitation were used as climatic predictors. Response function analysis is based on principal component regression and is particularly suitable for dendroclimatological studies because it reduces multicollinearity among monthly climate variables and identifies the climatic signals most strongly associated with tree growth [4,59]. For both monthly and seasonal response-function analyses, coefficient significance was assessed by using the bootstrap percentile procedure in DendroClim2002. To further assess the seasonal climatic controls on radial growth, seasonal variables were constructed based on the regional climatic regime and the growth phenology of *P. densata*. The season was designed to represent both lagged climatic effects before the current growing season and climatic conditions during different stages of the current season. Specifically, previous autumn (September–October of the previous year) was included to account for potential antecedent effects before winter; current spring (April–May) represented the early growing season before the full onset of the summer monsoon; current summer (June–August) represented the main warm and wet growing-season period; and current autumn (September–October) represented the late-growing season. Climate variables from September of the previous year to October of the current year were included in subsequent analyses to account for both current-year effects and potential lagged climatic influences [60].

To complement the response function analysis, redundancy analysis (RDA) was performed to identify the principal climatic factors explaining variation in radial growth across sites [61,62]. In the RDA, years were treated as observations, the three site-specific RES chronologies were used as response variables, and monthly climatic variables were used as explanatory variables. Significant predictors were selected by forward selection, and their explanatory power was assessed using Monte Carlo permutation tests with 999 permutations (*p* < 0.05). RDA was conducted in CANOCO 5.0. Data visualization was performed using ArcMap 10.8.1, Origin 2024, and R (version 4.3.2) [63], with figures generated in R using the ggplot2 package (version 4.0.2) [64].

For moving-window response analysis, the common intervals were 1902–2017 for YL, 1932–2017 for HB, and 1902–2015 for SK. A 32-year moving-forward window was applied to quantify temporal variation in the relationships between radial growth and monthly climate variables. The 32-year window length was selected to balance the need for sufficient sample size within each window and the ability to detect decadal-scale changes in growth–climate relationships. The year shown in the output represents the ending year of each window.

## 5. Conclusions

Our results indicate that spring moisture availability, summer thermal conditions, and antecedent winter precipitation are key climatic variables associated with radial growth of *P. densata* in northwestern Yunnan. At the lower-elevation YL and HB sites, radial growth was more sensitive to early-season moisture stress, as reflected in the negative association with May temperature and the positive association with May precipitation, while warmer conditions in August generally favored growth. In contrast, growth at the higher-elevation SK site was more responsive to late-growing-season temperature. The combined application of response function analysis and redundancy analysis provided complementary evidence for identifying important climatic factors associated with growth, whereas moving-window analysis further demonstrated that growth–climate relationships varied among sites, with site-specific changes in both the strength and direction of climatic responses through time. Together, these findings showed that the climatic sensitivity of *P. densata* in northwestern Yunnan was both spatially heterogeneous and temporally dynamic. Under continued regional warming, early-season drought stress may become an increasingly important constraint on radial growth. Therefore, assessments of potential forest responses to climate change and the development of adaptive management strategies should explicitly account for the spatial divergence and temporal non-stability of growth–climate relationships.

## Figures and Tables

**Table 1:** Statistical characteristics of the residual chronologies of *Pinus densata* at the three sites.

Statistical Characteristics	YL	HB	SK
No. (tree/cores)	25/46	24/48	27/48
Time span/A.D.	1879–2017	1931–2017	1819–2015
Mean sensitivity	0.14	0.09	0.08
Common interval analysis	1953–2015		
Standard deviation	0.12	0.08	0.07
Signal-to-noise ratio	9.52	28.40	30.04
Expressed population signal	0.91	0.97	0.97
Variance in first eigenvector (%)	37.77	44.62	41.18

YL, Yulong Snow Mountain; HB, Haba Snow Mountain; SK, Shika Snow Mountain.

**Table 2:** Seasonal response-function coefficients between residual chronologies and seasonal climatic variables.

Site	Mean Temperature				Precipitation			
	PAU	CSP	CSU	CAU	PAU	CSP	CSU	CAU
YL	−0.0493	−0.1328 *	0.0850	0.0172	−0.0183	0.1176 *	−0.0457	0.0024
SK	−0.0002	0.0069	−0.0114	0.0252	−0.0519	−0.0705	0.0033	−0.0541
HB	−0.0041	−0.1007 *	0.1136 *	−0.0516	−0.0164	0.0885 *	−0.0935 *	0.0300

Values are seasonal response-function coefficients calculated using DendroClim2002. Asterisks indicate significant coefficients at *p* < 0.05 based on bootstrap resampling. PAU, previous autumn; CSP, current spring; CSU, current summer; CAU, current autumn.

**Table 3:** Summary of the sampling sites.

Sampling Site	Longitude/E	Latitude/N	Altitude (m)	No. (Tree/Cores)
YL	100°14′04.62″	27°07′48.99″	3052	28/55
SK	99°36′33.70″	27°50′17.74″	3623	34/68
HB	100°08′50.34″	27°21′05.13″	3455	32/63

## Data Availability

The data supporting the findings of this study are included in the article and Supplementary Materials.

## Supplementary Materials

The following supporting information can be downloaded at: https://www.mdpi.com/article/10.3390/plants15152250/s1. Table S1. Forward selection results of RDA for the relationships between residual chronologies and climatic variables; Data S1: Minimal dataset supporting the findings of this study.

## References

[1] Piao S., Zhang X., Chen A., Liu Q., Lian X., Wang X., Peng S., Wu X. (2019). The impacts of climate extremes on the terrestrial carbon cycle: A review. Sci. China Earth Sci..

[2] Masson-Delmotte V., Zhai P., Pirani A., Connors S.L., IPCC (2021). Summary for policymakers. Climate Change 2021: The Physical Science Basis. Contribution of Working Group I to the Sixth Assessment Report of the Intergovernmental Panel on Climate Change.

[3] Chen Z., Zhou T., Chen X., Zhang W., Zuo M., Man W., Qian Y. (2023). Emergent constrained projections of mean and extreme warming in China. Geophys. Res. Lett..

[4] Fritts H.C. (1976). Tree Rings and Climate.

[5] He M., Yang B., Bräuning A., Rossi S., Ljungqvist F.C., Shishov V., Grießinger J., Wang J., Liu J., Qin C. (2019). Recent advances in dendroclimatology in China. Earth-Sci. Rev..

[6] Babst F., Bouriaud O., Poulter B., Trouet V., Girardin M.P., Frank D.C. (2019). Twentieth century redistribution in climatic drivers of global tree growth. Sci. Adv..

[7] Wilmking M., van der Maaten-Theunissen M., van der Maaten E., Scharnweber T., Buras A., Biermann C., Gurskaya M., Hallinger M., Lange J., Shetti R. (2020). Global assessment of relationships between climate and tree growth. Glob. Change Biol..

[8] Wei X., Peng J., Peng M., Li X., Cui J., Li J., Wei Y. (2024). Response of *Pinus tabuliformis* radial growth to climate factors in the north-south transition zone of China: Case study in the Jigong Mountain. Sci. Geogr. Sin..

[9] Li Q., Li J., Au T.F., Li T. (2025). From correlation to mechanism: A bibliometric analysis of dendrochronological research evolution on tree growth responses to climate change. Dendrochronologia.

[10] Rossi S., Deslauriers A., Gricar J., Seo J.W., Rathgeber C.B.K., Anfodillo T., Morin H., Levanic T., Oven P., Jalkanen R. (2008). Critical temperatures for xylogenesis in conifers of cold climates. Glob. Ecol. Biogeogr..

[11] Wang Z., Yang B., Deslauriers A., Bräuning A. (2015). Intra-annual stem radial increment response of Qilian juniper to temperature and precipitation along an altitudinal gradient in northwestern China. Trees.

[12] Zhang Q., Yuan R., Singh V.P., Xu C.-Y., Fan K., Shen Z., Wang G., Zhao J. (2022). Dynamic vulnerability of ecological systems to climate changes across the Qinghai-Tibet Plateau, China. Ecol. Indic..

[13] Zhang C., Qin D.-H., Zhai P.-M. (2023). Amplification of warming on the Tibetan Plateau. Adv. Clim. Change Res..

[14] Bian C., Liang X., Li B., Hu Z., Min X., Yue Z. (2025). The future climate change projections for the Hengduan Mountain region based on CMIP6 models. Sustainability.

[15] Fan Z.X., Bräuning A., Cao K.F. (2008). Tree-ring based drought reconstruction in the central Hengduan Mountains region (China) since AD 1655. Int. J. Climatol..

[16] Lin Y., Li M. (2024). The first-order difference data of tree-ring maximum latewood density in Northwest Yunnan can better reveal the interannual variation of temperature. Adv. Earth Sci..

[17] Panthi S., Bräuning A., Zhou Z.K., Fan Z.X. (2018). Growth response of *Abies georgei* to climate increases with elevation in the central Hengduan Mountains, southwestern China. Dendrochronologia.

[18] Zhang Y., Yin D., Tian K., Zhang W., He R., He W., Sun J., Liu Z. (2018). Radial growth responses of *Picea likiangensis* to climate variabilities at different altitudes in Yulong Snow Mountain, southwest China. Chin. J. Plant Ecol..

[19] Zhang W., Xiao D., Tian K., Chen G., He R., Zhang Y. (2017). Radial growth and climate response of three coniferous tree species at their upper altitudinal limits in Yulong Snow Mountain. Acta Ecol. Sin..

[20] Zhang Y., Yin D., Sun M., Wang H., Tian K., Xiao D., Zhang W. (2017). Variations of climate-growth response of major conifers at upper distributional limits in Shika Snow Mountain, Northwestern Yunnan Plateau, China. Forests.

[21] Peng X., Yang R., Yin Y., Panthi S., Xu T., Fu P., Ge S., Fan Z.-X. (2023). Radial growth response of *Pinus densata* to climatic factors in Baima Snow Mountain, Northwest Yunnan. Acta Ecol. Sin..

[22] Xie S., Zhang Y., Kang Y., Yan T., Yue H. (2024). The growth–climate relationships of three dominant subalpine conifers on the Baima Snow Mountain in the southeastern Tibetan Plateau. Plants.

[23] Wu Z.L. (1993). Preliminary analysis of the relationships among *Pinus yunnanensis*, *Pinus densata*, and *Pinus kesiya* var. langbianensis. J. Shanxi Norm. Univ. (Nat. Sci. Ed.).

[24] Zheng W.J., Fu L.G. (1978). Flora Reipublicae Popularis Sinicae.

[25] Du Y., Bao W. (2022). Research progress on natural geographic distribution, growth law and ecological adaptability of *Pinus densata*. J. Sichuan For. Sci. Technol..

[26] Du Y., Liu X., Zhang H.-Y., Ma S.-W., Bao W.-K. (2025). Community characteristics of *Pinus densata* alliance in China. Chin. J. Plant Ecol..

[27] Han D., Zhang J., Xu D., Liao Y., Bao R., Wang S., Chen S. (2024). Improving *Pinus densata* carbon stock estimations through remote sensing in Shangri-La: A nonlinear mixed-effects model integrating soil thickness and topographic variables. Forests.

[28] Yue H., Li J., Xie S., Chen H., Tian K., Sun M., Zhang D., Zhang Y. (2023). Relationships between climate variability and radial growth of *Larix potaninii* at the upper altitudinal limit in central Hengduan Mountain, southwestern China. Forests.

[29] Wu P., Wang L.-L., Huang L. (2006). A preliminary study on the tree-ring sensitivity to climate change of five endemic conifer species in China. Geogr. Res..

[30] Yang Y., Zhang M., Zhang L., Lu Q., Hong Y., Liu X. (2022). Different responses of radial growth of *Pinus tabulaeformis* to climate factors in the central and western Qinling Mountains. Acta Ecol. Sin..

[31] Ren P., Rossi S., Camarero J.J., Ellison A.M., Liang E., Peñuelas J. (2018). Critical temperature and precipitation thresholds for the onset of xylogenesis of Juniperus przewalskii in a semi-arid area of the north-eastern Tibetan Plateau. Ann. Bot..

[32] Sun L., Cai Y., Zhou Y., Shi S., Zhao Y., Gunnarson B.E., Jaramillo F. (2020). Radial growth responses to climate of *Pinus yunnanensis* at low elevations of the Hengduan Mountains, China. Forests.

[33] Li X., Liang E., Gričar J., Rossi S., Čufar K., Ellison A.M. (2017). Critical minimum temperature limits xylogenesis and maintains treelines on the southeastern Tibetan Plateau. Sci. Bull..

[34] Pu X., Lyu L. (2025). Tree growth and water-use efficiency at the Himalayan fir treeline and lower altitudes: Roles of climate warming and CO_2_ fertilization. Biogeosciences.

[35] Li T., He X.-Y., Chen Z.-J. (2014). Tree-ring growth responses of Mongolian oak (*Quercus mongolica*) to climate change in southern Northeast: A case study in Qianshan Mountains. Chin. J. Appl. Ecol..

[36] Shen J., Zaw Z., Huang X., Shang R., Yang R., Liu W., Fan Z., Su J. (2025). Context-dependent effects of drought severity and climate sensitivity on growth resilience of *Pinus yunnanensis* in Yunnan, SW China. Clim. Change.

[37] Zhao H., Wu J., Wang A., Guan D., Liu Y. (2022). Microtopography mediates the climate–growth relationship and growth resilience to drought of *Pinus tabulaeformis* plantation in the hilly site. Front. Plant Sci..

[38] Castagneri D., Fonti P., von Arx G., Carrer M. (2017). How does climate influence xylem morphogenesis over the growing season? Insights from long-term intra-ring anatomy in *Picea abies*. Ann. Bot..

[39] Li J., Song F., Jin Y., Yun R., Chen Z., Lyu Z., Zhao Y., Cui D. (2021). Critical temperatures controlling the phenology and radial growth of *Pinus sylvestris* var. mongolica on the southern margin of a cold temperate coniferous forest. Ecol. Indic..

[40] Körner C. (2012). Alpine Treelines: Functional Ecology of the Global High Elevation Tree Limits.

[41] Li J., Liu Z., Wang P., Yang R., Shi F., Deng J., Wang G., Shi S. (2024). Responses of radial growth of *Pinus wallichiana* to climate change in the Mount Everest region, Tibet. Chin. J. Appl. Ecol..

[42] Chen Q., Liu N., Bao G., Cheng X., Wang Y., He K., Zhang W., Wang G. (2025). Growth response of *Pinus tabuliformis* and *Abies fargesii* to climate factors in southern slope of central Qinling Mountains of China. Forests.

[43] Yan T., Kang Y., Xie S., Tao C., Li L., Li X., Wang Q., Zhang Y. (2025). Differences in growth responses to climate of three conifer species in Lugu Lake of northwestern Yunnan, southwestern China. Plants.

[44] Cao R., Yin D., Tian K., Xiao D., Li Z., Zhang X., Li Z., Zhang Y. (2020). Radial growth responses of *Abies georgei* and *Tsuga dumosa* to climate change at their upper elevational limits in Laojun Mountain, Lijiang. Acta Ecol. Sin..

[45] Keyimu M., Li Z., Fu B., Liu G., Zeng F., Chen W., Fan Z., Fang K., Wu X., Wang X. (2021). A 406-year non-growing-season precipitation reconstruction in the southeastern Tibetan Plateau. Clim. Past.

[46] Sun M., Li J., Cao R., Tian K., Zhang W., Yin D., Zhang Y. (2021). Climate-growth relations of *Abies georgei* along an altitudinal gradient in Haba Snow Mountain, Southwestern China. Forests.

[47] Peltier D.M.P., Ogle K. (2020). Tree growth sensitivity to climate is temporally variable. Ecol. Lett..

[48] Liu Y., Wang Y., Liu B., Liang E. (2024). Response of radial growth of *Pinus densata* forest to climate change in Milin City of Xizang, China. Quat. Sci..

[49] Feng A., Zhu Z., Zhu X., Zhang Q., Wang M., Li H., Wang Y., Wang Z., Sun P., Wang G. (2025). The multiple impacts of climate change and human activities on vegetation dynamics in Yunnan Province, China. Sustainability.

[50] Li W., Sun L., Chen K., Zhang Z., Chen J. (2023). Diversity and conservation of higher plants in Northwest Yunnan-Southeast Tibet. Glob. Ecol. Conserv..

[51] Fan Z.X., Bräuning A., Cao K.F. (2008). Annual temperature reconstruction in the central Hengduan Mountains, China, as deduced from tree rings. Dendrochronologia.

[52] Guo L.N., He Z.J., Long X.Z., Wang J.Z., Wang L.D., Li C.X. (2009). Characterization of soil occurrence and its systematic classification in Yulong Snow Mountain. Guangxi Agric. Sci..

[53] Xie S., Yan T., Sun X., Chen H., Sun M., Zhang Y. (2024). Radial growth response of *Pinus yunnanensis* to climate in high mountain forests of northwestern Yunnan, southwestern China. Front. For. Glob. Change.

[54] Grissino-Mayer H.D., Fritts H.C. (1997). The International Tree-Ring Data Bank: An enhanced global database serving the global scientific community. Holocene.

[55] Holmes R.L. (1983). Computer-assisted quality control in tree-ring dating and measurement. Tree-Ring Bull..

[56] Cook E.R. (1986). Users manual for program ARSTAN. Tree-Ring Chronologies of Western North America: California, Eastern Oregon and Northern Great Basin.

[57] Harris I., Osborn T.J., Jones P., Lister D. (2020). Version 4 of the CRU TS monthly high-resolution gridded multivariate climate dataset. Sci. Data.

[58] Biondi F., Waikul K. (2004). DENDROCLIM2002: A C++ program for statistical calibration of climate signals in tree-ring chronologies. Comput. Geosci..

[59] Fritts H.C., Blasing T.J., Hayden B.P., Kutzbach J.E. (1971). Multivariate techniques for specifying tree-growth and climate relationships and for reconstructing anomalies in paleoclimate. J. Appl. Meteorol..

[60] Fan Z.X., Bräuning A., Cao K.F., Zhu S.D. (2009). Growth–climate responses of high-elevation conifers in the central Hengduan Mountains, southwestern China. For. Ecol. Manag..

[61] ter Braak C.J.F., Šmilauer P. (2012). Canoco Reference Manual and User’s Guide: Software for Ordination (Version 5.0).

[62] van den Wollenberg A.L. (1977). Redundancy analysis an alternative for canonical correlation analysis. Psychometrika.

[63] R Core Team (2026). R: A Language and Environment for Statistical Computing.

[64] Wickham H. (2016). ggplot2: Elegant Graphics for Data Analysis.

