# Supplementary material for: Site-Specific Climatic Responses of Radial Growth in Pinus densata in Northwestern Yunnan, Southwestern China
Source: Plants (Basel). 2026 Jul 23;15(15):2250. doi: 10.3390/plants15152250 (PMC13468430; doi:10.3390/plants15152250)
Supplement: Supplementary file 1 [file plants-15-02250-s001.zip › plants-4372281-supplementary.pdf]

Supplementary Materials

Table S1. Forward selection results of RDA for the relationships between residual chronologies and climatic variables.

Variable	Explained variation (%)	Contribution (%)	Pseudo-F	p value
Current August mean temperature	7.6	16.1	5.3	0.008
Current May mean temperature	6.8	14.5	4.5	0.018
Previous December precipitation	5.0	10.6	3.6	0.012

Note: The selected variables together explained 19.4% of the total variation in the three residual chronologies, with an adjusted explained variation of 15.3%. Significance was assessed using permutation tests in Canoco 5.
